# The Relationship between Furin and Chronic Inflammation in the Progression of Cervical Intraepithelial Neoplasia to Cancer: A Cross-Sectional Study

**DOI:** 10.3390/cancers15194878

**Published:** 2023-10-07

**Authors:** Selim Afsar, Gulay Turan, Gurhan Guney, Gozde Sahin, Merve Aldıkactıoglu Talmac, Cigdem Usul Afsar

**Affiliations:** 1 Department of Obstetrics & Gynecology, School of Medicine, Balıkesir University, Cagis Yerleskesi, Bigadic Yolu 17. km, Balikesir 10145, Turkey; gurhanguney@yahoo.com; 2 Department of Pathology, School of Medicine, Balıkesir University, Balıkesir 10145, Turkey; gulaytr@yahoo.com.tr; 3 Department of Gynecologic Oncology, Health Sciences University, Istanbul 34668, Turkey; sahin.gozde1983@gmail.com (G.S.); drmrve@gmail.com (M.A.T.); 4 Department of Medical Oncology, Health Sciences University, Istanbul 34668, Turkey; cigdemusul@yahoo.com

**Keywords:** furin, cervical intraepithelial neoplasia, cervical cancer, neutrophil-to-lymphocyte ratio, monocyte-to-lymphocyte ratio, platelet-to-lymphocyte ratio, systemic immune-inflammatory index

## Abstract

**Simple Summary:**

For the first time, we demystified the relationship between furin and chronic inflammation while cervical intraepithelial neoplasia progresses to cancer. Although the mechanism of entry of HPV into the cell has not been conclusively determined, the cleavage of the minor capsid protein L2 by furin is a sine qua non for HPV infection. The expression of furin increased stepwise along with the progression of cervical dysplasia to cervical cancer. It could be considered a new pathologic biomarker that signifies a developing cervical cancer and denotes the severity of cervical intraepithelial neoplasia.

**Abstract:**

Objective: The current study aimed to delineate the relationship between furin and chronic inflammation while cervical intraepithelial neoplasia progresses to cancer. Study Design: This cross-sectional study included 81 women who required colposcopic examinations. The study groups were formed based on pathological results: Group I included women with cervical intraepithelial neoplasia (CIN) I (n = 30); Group II included women with CIN II-III (n = 28); and Group III included women with cervical cancer (CC) (n = 23). Furin, ki-67, and p16 levels were evaluated based on immunostaining intensity. The inflammatory indices were calculated in parallel with the literature from routine blood samples retrieved within one week before the procedure. Results: Furin expression gradually increased from CIN I to CIN II-III and from CIN II-III to CC, respectively (*p* < 0.001, *p* = 0.005). NLR, MLR, PLR, and SII were significantly higher in the CC group (*p* < 0.001). ROC curve analysis unveiled that NLR, MLR, PLR, and SII predicted the presence of CC with a cutoff value of 2.39 for NLR (sensitivity: 91.3%, specificity: 63.8%, AUROC: 0.79, *p* < 0.001); a cutoff value of 0.27 for MLR (sensitivity: 78.3%, specificity: 72.4%, AUROC: 0.77, *p* = 0.009); a cutoff value of 123 for PLR (sensitivity: 100%, specificity: 41.4%, AUROC: 0.70, *p* = 0.04); and a cutoff value of 747 for SII (sensitivity: 69.6%, specificity: 90.7%, AUROC: 0.71, *p* = 0.014). Conclusion: Furin expression increased gradually in parallel with the severity of cervical intraepithelial neoplasia. The inflammatory indices were higher in the presence of CC and denoted a good discrimination ability for predicting cervical cancer.

## 1. Introduction

Cervical cancer (CC) is the fourth most common cancer among women worldwide. According to Global Cancer Observatory (GLOBOCAN) 2020 data, there are approximately 600,000 new CC cases and 350,000 deaths per year [1]. It has become increasingly apparent that persistent high-risk human papillomavirus (HR-HPV) infection is the leading underlying cause in the pathogenesis of cervical intraepithelial neoplasia, which may eventually progress to cervical cancer if left untreated [2]. The high-risk human papillomaviruses are 16, 18, 31, 33, 35, 39, 45, 51, 52, 56, 58, and 59. HPV 16 and 18 are associated with approximately 70% of cervical cancer cases globally [3].

Generally, 90% of HPV infections in the cervical epithelium is cleared by the immune system in the following eighteen months. If it persists, high-risk human papillomaviruses integrate their early E6 and E7 oncoproteins into basal cervical epithelial cells’ genome, ultimately leading to cervical intraepithelial neoplasia [4]. This integration results in uncontrolled replication of human papillomavirus oncoproteins and the release of pro-inflammatory molecules, and both together eventually cause chronic inflammation and the degradation of the tumor suppressor gene p53. Therefore, chronic inflammation is responsible for the progression of cervical dysplasia to cancer and HPV-mediated cancers [5,6].

Although the mechanism of entry of HPV into the cell has not been conclusively determined, the first step involves the attachment of the major L1 capsid protein of HPV to heparan sulfate proteoglycans (HSPGs) found within the plasma membrane of keratinocytes [7]. In subsequent processes, the cleavage of the minor capsid protein L2 by furin is a sine qua non for HPV infection. The cleavage of viral particles by furin enables endosomal escape and provides a bypass mechanism for the first step of entry [8,9].

Furin participates in critical steps of the immune response by regulating T cell and macrophage functions via TGFβ1 synthesis and modulating immune checkpoint signaling via programmed death-1 (PD-1) synthesis. Intriguingly, furin regulates the cancer microenvironment in favor of cancer cells by enabling immune escape for cancer cells via accelerated depletion of cytotoxic T cells. Although the mechanisms underlying its diverse functions have not been elucidated in depth yet, furin plays a crucial role in the tumor immune microenvironment via regulating the functions of myeloid and lymphoid cells and their regulatory molecules [10,11,12,13,14].

Currently, the tumor microenvironment (TME) has been subjected to comprehensive studies, and it has been proven that inflammatory cells, such as neutrophils, lymphocytes, macrophages, and platelets, are critical components of the immune response to cancer [10,11,12,13,14,15]. The number of circulating inflammatory cells and platelets and their indices are the most prominent indicators of immune responses to cancer [14]. Even if the role of neutrophils in cancer is ambiguous, a high neutrophil-to-lymphocyte ratio (NLR) is related to a poorer prognosis for various cancers, such as breast cancer, colorectal cancer, malignant melanoma, and gynecological cancer [16,17]. Likewise, a high monocyte-to-lymphocyte ratio (MLR) and a high platelet-to-lymphocyte ratio (PLR) are also considered valuable prognostic markers in multiple cancers, such as head and neck, gastric, colorectal, and brain metastasis [18,19,20,21]. Recently, the systemic immune-inflammation index (SII), which takes into account the platelet count, has been regarded as a superior prognostic factor for solid tumors, such as esophageal, gynecological, and triple-negative breast cancer, and predicts the response to immunotherapy [22,23,24]. 

Circulating inflammatory cells and platelets are the most salient indicators of immune responses to cancer and indirectly reflect the tumor immune microenvironment status. On the other hand, furin alters the cancer microenvironment in favor of cancer cells by enabling immune escape. Therefore, we hypothesized that both of these factors could interact with each other in the progression of cervical intraepithelial neoplasia to cancer. We aimed to elucidate the relationship between furin and chronic inflammation in the progression of cervical intraepithelial neoplasia to cervical cancer.

## 2. Materials and Methods

### 2.1. Study Design

This cross-sectional study recruited 81 women admitted to the Gynecology Department of the Balıkesir University School of Medicine between January 2018 and 2023. Women aged 30–65 who needed a colposcopic examination based on abnormal PAP smear (Thin-prep) screening results or HR-HPV DNA positivity (Rotor-Gene Q, Qiagen, Hilden, Germany) participated in the study. The participants were managed according to the current American Society for Colposcopy and Cervical Pathology (ASCCP) guidelines [25]. Participants either followed-up or underwent surgery consistent with their colposcopic biopsy results. 

The study groups were formed based on the pathological results as follows. Women with cervical intraepithelial neoplasia (CIN) 1 biopsy results (Group I, n = 30) were regularly followed; however, those with CIN 2 and 3 (Group II, n = 28) underwent a conization operation. Women with squamous CC (Group III, n = 23) underwent either a simple or radical hysterectomy based on their stage (Table 1). Routine blood samples were retrieved within one week before biopsy, conization, or surgery.

Women with acute infection and chronic inflammatory diseases (n = 17), diabetes (n = 13), a history of cancer (n = 3), vaginitis (n = 42), cervicitis (n = 7), and other sexually transmitted infections (n = 2), or receiving radiotherapy (n = 1), chemotherapy (n = 2), or anti-inflammatory or immunosuppressive agents (n = 18) were excluded from the study (n = 102). The STROBE (The Reporting of Observational Studies in Epidemiology) guidelines were followed [26].

### 2.2. Immunohistochemistry & Evaluation

Standardized tissue preparation protocols were followed during the histopathological examination of cervical tissues as previously described [27]. Subsequently, anti-furin (1:100; Santa Cruz Biotechnology Inc., Dallas, TX, USA), anti-ki-67 (1:100; Santa Cruz Biotechnology Inc., Dallas, TX, USA), and anti-p16 (1:50; Santa Cruz Biotechnology Inc., Dallas, TX, USA) antibodies were applied. All slides were examined and scored concurrently by two experienced pathologists who were blinded to the clinical diagnoses. Using an image capture system, the furin expression in the study groups was randomly compared based on immunohistochemistry (IHC) staining intensity.

**Figure 1:**
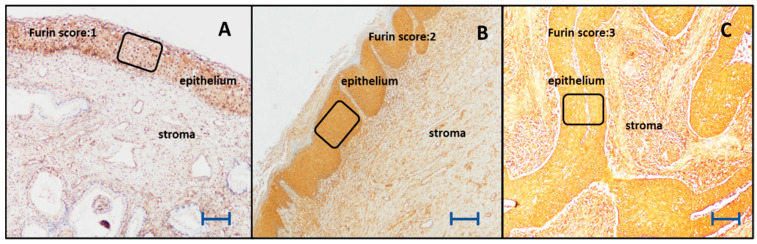
The expression levels of furin in the cervical epithelium (×200). The scale bar indicates 1 mm. (**A**) Weak furin expression in the cervical epithelium of a woman with low-grade cervical dysplasia (furin score 1). (**B**) Moderate furin expression in the cervical epithelium of a woman with high-grade cervical dysplasia (furin score 2). (**C**) Strong furin expression in the cervical epithelium of a woman with squamous cervical cancer (furin score 3).

The IHC staining intensity of furin, ki-67, and p16 was evaluated using a scoring system based on the staining ratio of the cervical cells. The furin scores were as follows: 10–40 staining 1(+); 41–70% staining 2(+); and >71% staining 3(+) (Figure 1A–C, respectively). For ki-67, the intensity score was graded as follows: 10–30% staining 1(+); 31–50% staining 2(+); 51–70%, 3(+); and ≥71%, 4(+). For p16, block-positive staining was scored 1(+); on the other hand, equivocal or negative staining was scored 0.

### 2.3. Indices

NLR, MLR, and PLR were calculated: the absolute number of neutrophils, platelets, and monocytes was divided by the absolute number of lymphocytes. SII was calculated using the following formula: SII = platelet count × neutrophil count/lymphocyte count [15].

### 2.4. Statistics

Statistical and power analyses were performed using open-source Jamovi statistical software (version 2.3.21) and G* Power software (version 3.1.9.7). According to the literature, the minimum sample size was calculated as twenty-two per group based on an α error of 0.01 and power of 0.80. The distribution and homogeneity of the groups were evaluated by skewness, kurtosis, Levene’s test, and the Kolmogorov–Smirnov test. 

The Kruskal–Wallis test was applied to nonparametric group variables, and pairwise comparisons were made using the Dwass–Steel–Critchlow–Fligner test. One-way ANOVA and Tukey’s post hoc tests were applied to the parametric group variables. Partial correlation analyses were performed using either Pearson or Spearman coefficients, for variables with normal and abnormal distributions, respectively. A receiver operating characteristic (ROC) curve was plotted to denote the performance of inflammatory indices in discriminating the CC from cervical intraepithelial neoplasia. Statistical significance was set at *p* < 0.05.

## 3. Results

This cross-sectional study reviewed the cervical pathology and laboratory results of 81 women. The mean age of the groups was 44.5 ± 13.7 years. There was a statistically significant difference between the CIN II-III and CC groups in the context of age (39.5 ± 12.9 years vs. 50.2 ± 13.7 years, respectively, *p* < 0.05); otherwise, the mean ages were similar between groups (Table 1).

Furin expression gradually increased from CIN I to CIN II-III and from CIN II-III to CC, respectively (*p* < 0.001, *p* = 0.005). ki-67 and p16 expression differed significantly between the study groups (*p* < 0.001), except for CIN II-III and CC (*p* = 0.817 and *p* = 0.771, respectively) (Table 1). The pathological results of the participants are summarized in Table 1.

There was a slightly higher WBC count in the CC group than in the CIN I group (*p* = 0.012) (Table 2). NLR, MLR, PLR, and SII were significantly higher in the CC group (*p* < 0.001). However, these were similar between CIN I and CIN II-III groups (Table 2). The laboratory results of the participants are summarized in Table 2.

The expression of furin was moderately correlated with NLR, MLR, PLR, and SII (rho: 0.57, *p* < 0.001; rho: 0.43, *p* < 0.001; rho: 0.38, *p* < 0.001; rho: 0.68, *p* < 0.001; respectively), and SII was strongly correlated with NLR and PLR (rho: 0.92 *p* < 0.001; rho: 0.70 *p* < 0.001; respectively) (Table 3). Table 3 summarizes the correlation analysis of furin and SII with the inflammatory indices.

**Figure 2:**
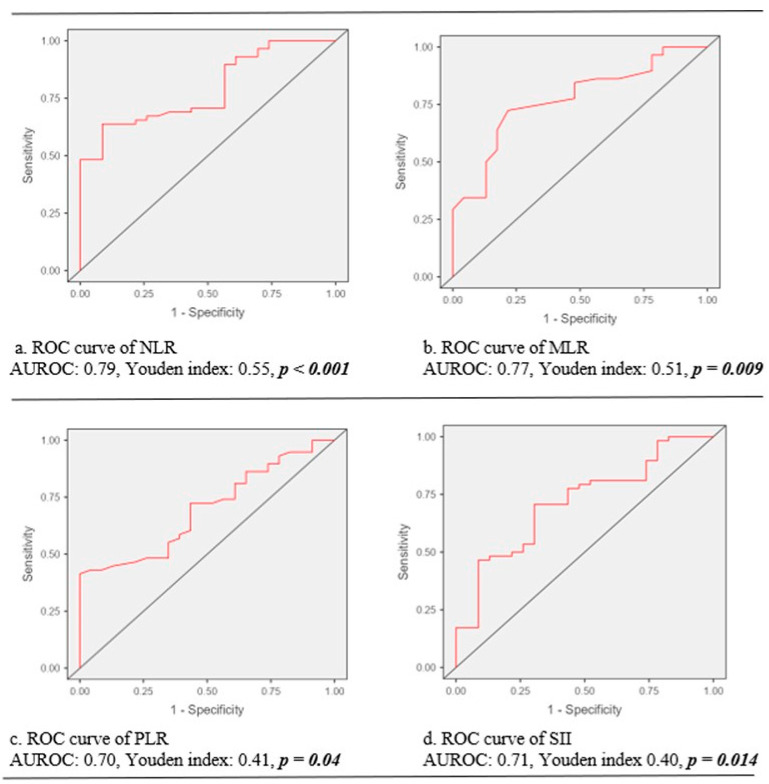
The predictive performance of inflammatory indices for cervical cancer. Note. ROC, receiver operating characteristic curve; AUROC, the area under the curve of receiver operating characteristic curve; NLR: neutrophil-to-lymphocyte ratio; MLR, monocyte-to-lymphocyte ratio; PLR, platelet-to-lymphocyte ratio; SII, systemic immune-inflammation index.

The ROC curve analysis unveiled that NLR, MLR, PLR and SII predicted the presence of CC with a cutoff value of 2.39 for NLR (sensitivity: 91.3%, specificity: 63.8%, AUROC: 0.79, Youden index: 0.55, *p* < 0.001); a cutoff value of 0.27 for MLR (sensitivity: 78.3%, specificity: 72.4%, AUROC: 0.77, Youden index: 0.51, *p* = 0.009); a cutoff value of 123 for PLR (sensitivity: 100%, specificity: 41.4%, AUROC: 0.70, Youden index: 0.41, *p* = 0.04); and a cutoff value of 747 for SII (sensitivity: 69.6%, specificity: 90.7%, AUROC: 0.71, Youden index: 0.40, *p* = 0.014) (Figure 2).

## 4. Discussion

This cross-sectional study evaluated the pathological and inflammatory markers of women with cervical epithelial neoplasia and cancer to elucidate the relationship between furin and inflammation in the progression of cervical intraepithelial neoplasia to cancer. The expression of furin increased stepwise along with the advancement of cervical dysplasia to cervical cancer, similar to p16 and ki-67. On the other hand, inflammatory indices were elevated only in women with cervical cancer but not in those with cervical dysplasia. Also, the inflammatory indices displayed good performance for discriminating CC from cervical intraepithelial neoplasia.

This study unveiled that the expression of furin is elevated in cervical cancer. Similarly, amplified furin expression has been revealed in gynecologic malignancies, especially in cervical and breast cancer, based on the Cancer Genome Atlas (TCGA) studies. It is considered either a poor prognostic marker or a proto-oncogene [28,29,30]. 

Nonetheless, we could not find any papers in the literature that argued for the importance of furin in cervical preinvasive conditions; this study elucidated that a gradual increase in furin correlated with the progression of cervical intraepithelial neoplasia to cervical cancer. It could be considered a new pathologic biomarker that signifies a developing cervical cancer and denotes the severity of cervical intraepithelial neoplasia; however, it needs to be supported by more studies [31]. 

Although the interpretation of p16 staining is still problematic and subjective, generally, p16 positivity and the high expression of ki-67 are desirable for diagnosing cervical intraepithelial neoplasia. This study confirmed the predictive role of p16/ki-67 staining in the neoplastic progression of cervical intraepithelial neoplasia, similar to that reported in the literature [32]. It also indicated that p16 was diffusely and firmly positive in a small number of low-grade cervical intraepithelial neoplasias (11%) and a moderate number of high-grade cervical intraepithelial neoplasias (30%) and cervical cancers (22%), similar to a study by Liu et al. [33]. It was proposed that the methylation of the p16 gene, which is particularly common in cervical cancer, might reduce the expression of p16 in cervical cancer patients, as might be the case in our study [34]. Our study found that ki-67 showed moderate staining extending from the basal and parabasal layers to half of the cervical epithelium in the low-grade cervical intraepithelial lesions. On the other hand, high-grade cervical intraepithelial lesions and cervical cancer showed intense ki-67 staining in all layers of the cervical epithelium. Our results are in line with a study by Chen et al. [4]. 

This study uncovered that, even if inflammatory indices (NLR, MLR, PLR, and SII) are raised in women with cervical cancer, they stayed in the normal range in women with cervical intraepithelial neoplasia. The underlying reason might be the invasion of cervical tissue in patients with cervical dysplasia, which is mainly by lymphocytes and M1-type macrophages [35,36,37]. It was demonstrated in the cervical tissue and serum of patients with cervical dysplasia and cancer that Th2, Th17, and Treg cells were significantly increased, and Th1 cells were significantly decreased [35]. It was also reported that dysplastic cervical tissue was infiltrated predominantly by M1-type macrophages; conversely, in cervical cancer tissue, M2-type, also known as tumor-associated, macrophages were prevalent [36]. It was concluded that persistent HR-HPV infection causes chronic Th2-type inflammation and facilitates tumor progression [38]. 

Additionally, the inflammatory indices in this study displayed good discrimination performance for discriminating CC from cervical intraepithelial neoplasia. The literature indicates that these inflammatory indices could be independently used to distinguish the preinvasive lesions in the larynx, cervix, and endometrium from their invasive counterparts [39,40,41]. Also, NLR, MLR, PLR, and SII could be used to predict certain cancer prognoses [16,17,18,19,20,21,22,23,24]. 

Although this study parallels the literature in differentiating preinvasive and malignant conditions, it opposes Xu and colleagues’ study because they claimed that inflammatory indices could predict the severity of cervical intraepithelial neoplasia [39,40,41,42]. The laudable study of Li and colleagues disclosed the mystery of cervical TME in which HSIL tissue displayed amplified infiltration by lymphocytes and M1-like macrophages [37]. Due to this reason, it could be speculated that the inflammatory indices are expected to be low in cervical intraepithelial neoplasia. In addition, it was denoted that furin orchestrates a myriad of pathways in cancer and inflammatory cells that can ultimately hamper immune cell infiltration into the TME [43]. 

## 5. Conclusions

The current study revealed that furin expression increases gradually in parallel with the severity of cervical intraepithelial neoplasia. The inflammatory indices (NLR, MLR, PLR, and SII) were higher in women with cervical cancer but similar between in the dysplasia groups. Inflammatory indices demonstrated a good discrimination ability for the prediction of cervical cancer.

### The Limitations of the Study

In addition to its retrospective nature, inflammatory indices are influenced by age, diet, activity level, and menstrual status. The limitations of this study include the absence of TILs, PD-L1, and furin mRNA expression in inflammatory and cervical cells in the study groups, which would make it easier to conclude the relationship between inflammation and cancer. As previously mentioned, little research has been conducted on the relationship between furin and preinvasive cervical disease; as a result, it could be assumed that there will be backlash to this study’s conclusion.

## Figures and Tables

**Table 1:** The pathological results of participants in the study groups.

	Participants (n = 81)	CIN I (n=30)	CIN II-III (n = 28)	CC (n = 23)	*p*
Age (year) ± SD	44.5 ± 13.8	44.7 ± 13.2	39.5 ± 12.9	50.2 ± 13.7	***0*** ***.020***
Furin					***<0.001***
0	17%	16%	1%	0%	
1	44%	21%	17%	6%	
2	30%	0%	16%	14%	
3	9%	0%	0%	9%	
ki-67					***<0.001***
1	23%	21%	2%	0%	
2	19%	11%	4%	4%	
3	22%	1%	11%	10%	
4	36%	4%	17%	15%	
p16					***<0.001***
0	37%	26%	5%	6%	
1	63%	11%	30%	22%	

Note. SD: standard deviation. Age is expressed as the mean ± SD.

**Table 2:** The laboratory results and inflammatory indices of women in the study groups.

	Participants (n = 81)	CIN I (n = 30)	CIN II-III (n = 28)	CC (n = 23)	*p*
WBC 10^3^/μL)	7.8 ± 2.1	6.2 ± 1.5	7.6 ± 2.0	8.8 ± 2.7 *	0.012 *
PLT (10^3^/μL)	295 ± 66	312 ± 76	283 ± 60	281 ± 64	*0.265*
NLR	3.08 ± 4.96	2.26 ± 0.74	2.33 ± 0.77	5.05 ± 9.07 ***	<0.001 ***
MLR	0.29 ± 0.25	0.26 ± 0.07	0.22 ± 0.06	0.42 ± 0.43 ***	<0.001 ***
PLR	146 ± 100	146 ± 48	125 ± 30	163 ± 153 ***	<0.001 ***
SII	900 ± 1412	693 ± 231	665 ± 300	1353 ± 2268 ***	<0.001 ***

Note. * *p* < 0.05, *** *p* < 0.001. WBC, white blood cell; PLT, platelet; NLR, neutrophil-to-lymphocyte ratio; MLR, monocyte-to-lymphocyte ratio; PLR, platelet-to-lymphocyte ratio; SII, systemic immune-inflammation index.

**Table 3:** Correlation analysis of furin and SII with other biochemical parameters.

	Furin		SII	
	CC	*p*	CC	*p*
NLR	0.57	*** <0.001 ***	0.92	*** <0.001 ***
MLR	0.43	*** <0.001 ***	0.49	*** <0.001 ***
PLR	0.38	*** <0.001 ***	0.70	*** <0.001 ***
SII	0.68	*** <0.001 ***	N/A	

Note. CC, correlation coefficient; N/A, not applicable; NLR, neutrophil-to-lymphocyte ratio; MLR, monocyte-to-lymphocyte ratio; PLR, platelet-to-lymphocyte ratio; SII, systemic immune-inflammation index.

## Data Availability

Data are available upon reasonable request from the corresponding author.

## References

[1] Singh D., Vignat J., Lorenzoni V., Eslahi M., Ginsburg O., Lauby-Secretan B., Arbyn M., Basu P., Bray F., Vaccarella S. (2023). Global estimates of incidence and mortality of cervical cancer in 2020, a baseline analysis of the WHO Global Cervical Cancer Elimination Initiative. Lancet Glob. Health.

[2] Chu D., Liu T., Yao Y. (2023). Implications of viral infections and oncogenesis in uterine cervical carcinoma etiology and pathogenesis. Front. Microbiol..

[3] Evans A.M., Salnikov M., Gameiro S.F., Maleki Vareki S., Mymryk J.S. (2022). HPV-Positive and -Negative Cervical Cancers Are Immunologically Distinct. J. Clin. Med..

[4] Chen R., Zhang R., Zhang M., Liu S., Xie M., Yang Z., Shi Q., Chen H., Xiong H., Wang N. (2023). CIN grades possessing different HPV RNA location patterns and RNAscope is a helpful tool for distinguishing squamous intraepithelial lesions in difficult cervical cases. Diagn. Pathol..

[5] Hemmat N., Bannazadeh Baghi H. (2019). Association of human papillomavirus infection and inflammation in cervical cancer. Pathog. Dis..

[6] Lifsics A., Cistjakovs M., Sokolovska L., Deksnis R., Murovska M., Groma V. (2023). The Role of the p16 and p53 Tumor Suppressor Proteins and Viral HPV16 E6 and E7 Oncoproteins in the Assessment of Survival in Patients with Head and Neck Cancers Associated with Human Papillomavirus Infections. Cancers.

[7] Kines R.C., Schiller J.T. (2022). Harnessing Human Papillomavirus’ Natural Tropism to Target Tumors. Viruses.

[8] Cruz L., Biryukov J., Conway M.J., Meyers C. (2015). Cleavage of the HPV16 Minor Capsid Protein L2 during Virion Morphogenesis Ablates the Requirement for Cellular Furin during De Novo Infection. Viruses.

[9] Wang J.W., Roden R.B. (2013). L2, the minor capsid protein of papillomavirus. Virology.

[10] Derynck R., Turley S.J., Akhurst R.J. (2021). TGFβ biology in cancer progression and immunotherapy. Nat. Rev. Clin. Oncol..

[11] Tomé M., Pappalardo A., Soulet F., López J.J., Olaizola J., Leger Y., Dubreuil M., Mouchard A., Fessart D., Delom F. (2019). Inactivation of proprotein convertases in T cells Inhibits PD-1 expression and creates a favorable immune microenvironment in colorectal cancer. Cancer Res..

[12] DeNardo D.G., Ruffell B. (2019). Macrophages as regulators of tumour immunity and immunotherapy. Nat. Rev. Immunol..

[13] Rose M., Duhamel M., Rodet F., Salzet M. (2021). The role of proprotein convertases in the regulation of the function of immune cells in the oncoimmune response. Front. Immunol..

[14] Cordova Z.M., Grönholm A., Kytölä V., Taverniti V., Hämäläinen S., Aittomäki S., Niininen W., Junttila I., Ylipää A., Nykter M. (2016). Myeloid cell-expressed proprotein convertase furin attenuates inflammation. Oncotarget.

[15] Huang H., Liu Q., Zhu L., Zhang Y., Lu X., Wu Y., Liu L. (2019). Prognostic Value of Preoperative Systemic Immune-Inflammation Index in Patients with Cervical Cancer. Sci. Rep..

[16] Xiong S., Dong L., Cheng L. (2021). Neutrophils in cancer carcinogenesis and metastasis. J. Hematol. Oncol..

[17] Cupp M.A., Cariolou M., Tzoulaki I., Aune D., Evangelou E., Berlanga-Taylor A.J. (2020). Neutrophil to lymphocyte ratio and cancer prognosis: An umbrella review of systematic reviews and meta-analyses of observational studies. BMC Med..

[18] Kumarasamy C., Tiwary V., Sunil K., Suresh D., Shetty S., Muthukaliannan G.K., Baxi S., Jayaraj R. (2021). Prognostic Utility of Platelet-Lymphocyte Ratio, Neutrophil-Lymphocyte Ratio and Monocyte-Lymphocyte Ratio in Head and Neck Cancers: A Detailed PRISMA Compliant Systematic Review and Meta-Analysis. Cancers.

[19] Hirahara N., Matsubara T., Kaji S., Hayashi H., Sasaki Y., Kawakami K., Hyakudomi R., Yamamoto T., Tajima Y. (2023). Novel inflammation-combined prognostic index to predict survival outcomes in patients with gastric cancer. Oncotarget.

[20] Rossi S., Basso M., Strippoli A., Schinzari G., D’Argento E., Larocca M., Cassano A., Barone C. (2017). Are Markers of Systemic Inflammation Good Prognostic Indicators in Colorectal Cancer?. Clin. Color. Cancer.

[21] Picarelli H., Yamaki V.N., Solla D.J.F., Neville I.S., Santos A.G.D., de Freitas B.S.A.G., Diep C., Paiva W.S., Teixeira M.J., Figueiredo E.G. (2022). The preoperative neutrophil-to-lymphocyte ratio predictive value for survival in patients with brain metastasis. Arq. Neuropsiquiatr..

[22] Li X., Zhang S., Lu J., Li C., Li N. (2022). The prognostic value of systemic immune-inflammation index in surgical esophageal cancer patients: An updated meta-analysis. Front. Surg..

[23] Ji Y., Wang H. (2020). Prognostic prediction of systemic immune-inflammation index for patients with gynecological and breast cancers: A meta-analysis. World J. Surg. Oncol..

[24] Tian B.W., Yang Y.F., Yang C.C., Yan L.J., Ding Z.N., Liu H., Xue J.-S., Dong Z.-R., Chen Z.-Q., Hong J.-G. (2022). Systemic immune-inflammation index predicts prognosis of cancer immunotherapy: Systemic review and meta-analysis. Immunotherapy.

[25] Perkins R.B., Guido R.S., Castle P.E., Chelmow D., Einstein M.H., Garcia F., Huh W.K., Kim J.J., Moscicki A.-B., Nayar R. (2020). 2019 ASCCP Risk-Based Management Consensus Guidelines Committee. 2019 ASCCP Risk-Based Management Consensus Guidelines for Abnormal Cervical Cancer Screening Tests and Cancer Precursors. J. Low. Genit. Tract Dis..

[26] von Elm E., Altman D.G., Egger M., Pocock S.J., Gøtzsche P.C., Vandenbroucke J.P., STROBE Initiative (2007). The Strengthening the Reporting of Observational Studies in Epidemiology (STROBE) statement: Guidelines for reporting observational studies. Lancet.

[27] Sancakli Usta C., Altun E., Afsar S., Bulbul C.B., Usta A., Adalı E. (2020). Overexpression of programmed cell death ligand 1 in patients with CIN and its correlation with human papillomavirus infection and CIN persistence. Infect. Agent Cancer.

[28] Zehir A., Benayed R., Shah R.H., Syed A., Middha S., Kim H.R., Srinivasan P., Gao J., Chakravarty D., Devlin S.M. (2017). Mutational landscape of metastatic cancer revealed from prospective clinical sequencing of 10,000 patients. Nat. Med..

[29] Huo X., Zhou X., Peng P., Yu M., Zhang Y., Yang J., Cao D., Sun H., Shen K. (2021). Identification of a six-gene signature for predicting the overall survival of cervical cancer patients. Onco Targets Ther..

[30] Li Y., Chu J., Li J., Feng W., Yang F., Wang Y., Zhang Y., Sun C., Yang M., Vasilatos S.N. (2018). Cancer/testis antigen-Plac1 promotes invasion and metastasis of breast cancer through Furin/NICD/PTEN signaling pathway. Mol. Oncol..

[31] López de Cicco R., Watson J.C., Bassi D.E., Litwin S., Klein-Szanto A.J. (2004). Simultaneous expression of furin and vascular endothelial growth factor in human oral tongue squamous cell carcinoma progression. Clin. Cancer Res..

[32] Mandal R., Ghosh I., Banerjee D., Mittal S., Muwonge R., Roy C., Panda C., Vernekar M., Frappart L., Basu P. (2020). Correlation between p16/Ki-67 Expression and the Grade of Cervical Intraepithelial Neoplasias. Int. J. Gynecol. Pathol..

[33] Liu J., Su S., Liu Y. (2022). The value of Ki67 for the diagnosis of LSIL and the problems of p16 in the diagnosis of HSIL. Sci. Rep..

[34] Wang F.L., Yang Y., Liu Z.Y., Qin Y., Jin T. (2017). Correlation between methylation of the p16 promoter and cervical cancer incidence. Eur. Rev. Med Pharmacol. Sci..

[35] Lin W., Niu Z., Zhang H., Kong Y., Wang Z., Yang X., Yuan F. (2019). Imbalance of Th1/Th2 and Th17/Treg during the development of uterine cervical cancer. Int. J. Clin. Exp.Pathol..

[36] Jayshree R.S. (2021). The Immune Microenvironment in Human Papilloma Virus-Induced Cervical Lesions-Evidence for Estrogen as an Immunomodulator. Front. Cell. Infect. Microbiol..

[37] Li C., Hua K. (2022). Dissecting the Single-Cell Transcriptome Network of Immune Environment Underlying Cervical Premalignant Lesion, Cervical Cancer and Metastatic Lymph Nodes. Front. Immunol..

[38] Feng Q., Wei H., Morihara J., Stern J., Yu M., Kiviat N., Hellstrom I., Hellstrom K.E. (2012). Th2-type inflammation promotes the gradual progression of HPV-infected cervical cells to cervical carcinoma. Gynecol. Oncol..

[39] Tas M., Yavuz A., Ak M., Ozcelik B. (2019). Neutrophil-to-Lymphocyte Ratio and Platelet-to-Lymphocyte Ratio in Discriminating Precancerous Pathologies from Cervical Cancer. J. Oncol..

[40] Kara A., Guven M., Demir D., Yilmaz M.S., Gundogan M.E., Genc S. (2019). Are calculated ratios and red blood cell and platelet distribution width really important for laryngeal cancer and precancerous larynx lesions?. Niger. J. Clin. Pract..

[41] Acmaz G., Aksoy H., Unal D., Ozyurt S., Cingillioglu B., Aksoy U., Muderris I. (2014). Are neutrophil/lymphocyte and platelet/lymphocyte ratios associated with endometrial precancerous and cancerous lesions in patients with abnormal uterine bleeding?. Asian Pac. J. Cancer Prev..

[42] Xu L., Song J. (2021). Elevated neutrophil-lymphocyte ratio can be a biomarker for predicting the development of cervical intraepithelial neoplasia. Medicine.

[43] He Z., Khatib A.M., Creemers J.W.M. (2022). The proprotein convertase furin in cancer: More than an oncogene. Oncogene.

